# Predictors of hospital mortality and multidrug-resistant pathogens in hospitalized pneumonia patients residing in the community

**DOI:** 10.1016/j.heliyon.2023.e22303

**Published:** 2023-11-15

**Authors:** Tomohiko Ukai, Takaya Maruyama, Shinichi Tomioka, Takumi Fukui, Shinya Matsuda, Kiyohide Fushimi, Hiroyasu Iso

**Affiliations:** a Department of Epidemiology and Clinical Research, The Research Institute of Tuberculosis, 3-1-24, Matsuyama, Kiyose City, Tokyo, 204-8553, Japan; b Division of Public Health, Osaka Institute of Public Health, 1-3-69 Nakamichi, Higashinari-ku, Osaka, Osaka 537-0025, Japan; c Mie Prefectural Ichishi Hospital, Tsu, Mie, 513-3133, Japan; d Call Medical Clinic Fukuoka, Munakata, Fukuoka 811-3516, Japan; e Department of Preventive Medicine and Community Health, School of Medicine, University of Occupational and Environmental Health, 1-1, Iseigaoka, Yahatanishiku, Kitakyushu, 807-8555, Japan; f Department of Health Policy and Informatics, Tokyo Medical and Dental University, 1-5-45, Yushima, Bunkyoku, Tokyo, 113-8510, Japan; g Institute for Global Health Policy Research, National Center for Global Health and Medicine, 1-21-1 Toyama Shinjuku-ku, Tokyo, 162-8655, Japan

**Keywords:** Community-acquired pneumonia, Healthcare-associated pneumonia, Antibiotic therapy, Guidelines, Predictors, Mortality, Multidrug-resistant pathogens

## Abstract

**Background and objective:**

The 2019 ATS/ADSA guidelines for adult community-acquired pneumonia (CAP) eliminated healthcare-associated pneumonia (HCAP) and considered it to be a form of CAP. This concept, however, was based on studies with relatively small sample sizes.

**Methods:**

We investigated the risk factors of 30-day mortality, and methicillin-resistant Staphylococcus aureus (MRSA) and *Pseudomonas aeruginosa* infections in patients with pneumonia coming from the community using the Diagnosis Procedure Combination database, a nationwide discharge database of acute care hospitals. Furthermore, we compared these factors between CAP and HCAP.

**Results:**

A total of 272,337 patients aged ≥20 years with pneumonia were grouped into 145,082 CAP patients and 127,255 HCAP patients. The 30-day mortality rate (8.9 % vs.3.3 %), MRSA infection (2.4 % vs. 1.4 %), and Pseudomonas aeruginosa infection (1.6 % vs. 1.0 %) were significantly higher in HCAP than in CAP patients. Multivariable logistic regression analysis showed that 12 of 13 identified predictors of mortality (i.e., high age, male, underweight, non-ambulatory status, bedsore, dehydration, respiratory failure, consciousness disturbance, hypotension, admitted in critical care, comorbidity of heart failure, and chronic obstructive pulmonary disease) were identical in CAP and HCAP patients. Similarly, five of six distinct risk factors for MRSA infection, and three of three for Pseudomonas aeruginosa infection were identical between the patients.

**Conclusion:**

The risk factors for mortality and MRSA or *Pseudomonas aeruginosa* infection were almost identical in patients with CAP and HCAP. The assessment of individual risk factors for mortality and MRSA or *Pseudomonas aeruginosa* infection in CAP and abandoning categorization as HCAP can improve and simplify empiric therapy.

## 1 Introduction

Pneumonia is a common condition that remains one of the leading causes of death worldwide [1]. To achieve better outcomes, accurate assessment and classification of patients with pneumonia are imperative to provide appropriate treatment. To achieve this goal, the American Thoracic Society/Infectious Diseases Society of America (ATS/ADSA) guidelines in 2005 and 2007 introduced healthcare-associated pneumonia (HCAP), in addition to community-acquired pneumonia (CAP), as new classifications of pneumonia that need to be treated as hospital-acquired pneumonia (HAP) [2,3].

While research from the United States has shown a higher prevalence of multidrug-resistant (MDR) pathogens in HCAP patients than in CAP patients [4,5], many studies report that HCAP patients have a lower prevalence of MDR pathogens than HAP patients, and that HCAP patients have a similar prevalence of MDR compared to CAP patients [[6], [7], [8]]. This misestimation of MDR pathogens can cause the overuse of antibiotics leading to adverse outcomes [9,10]. Additionally, a systematic review and meta-analysis by Chalmers et al. reported that the evidence supporting the concept of HCAP is based on low-quality research, and may not accurately identify MDR pathogens [11].

Following this debate, the dependent concept of HCAP was eliminated from the 2016 ATS/IDSA guidelines for nosocomial pneumonia and the 2019 guidelines for CAP, and it was considered a form of CAP [12,13]. This suggests that exposure to healthcare is not necessarily the determining factor in deciding the treatment strategy, rather the accurate assessment of individual risks for carrying MDR pathogens and mortality is even more important.

Considering the above, we aimed to update and establish the risk factors for mortality and MDR pneumonia, dividing them into Methicillin-resistant *Staphylococcus aureus* (MRSA) and *Pseudomonas aeruginosa* infections*,* and to re-evaluate the classification of CAP and HCAP using the Diagnosis Procedure Combination (DPC) database, which comprise all pneumonia admissions in Japan and their detailed clinical and background information.

## 2 Methods

### 2.1 Data source

We retrospectively studied patients who were admitted with pneumonia between 2016 and 2017 using records from the DPC database. Detailed information on the DPC database has been described elsewhere [14,15] and is provided in the online supplement. In brief, the database provides clinical and procedural information on all 82 university hospitals and other acute care hospitals in Japan, covering approximately eight million admissions per year. Participating hospitals have an incentive to provide accurate data under the public insurance system. Physicians are responsible for ensuring the accuracy of clinical information.

Study approval was obtained from the Institutional Review Board of the Ethical Committee for Epidemiology of Hiroshima University (E−2104). Due to the anonymous nature of the data, the requirement for patient informed consent was waived.

### 2.2 Study population and definitions of pneumonia

**Fig. 1:**
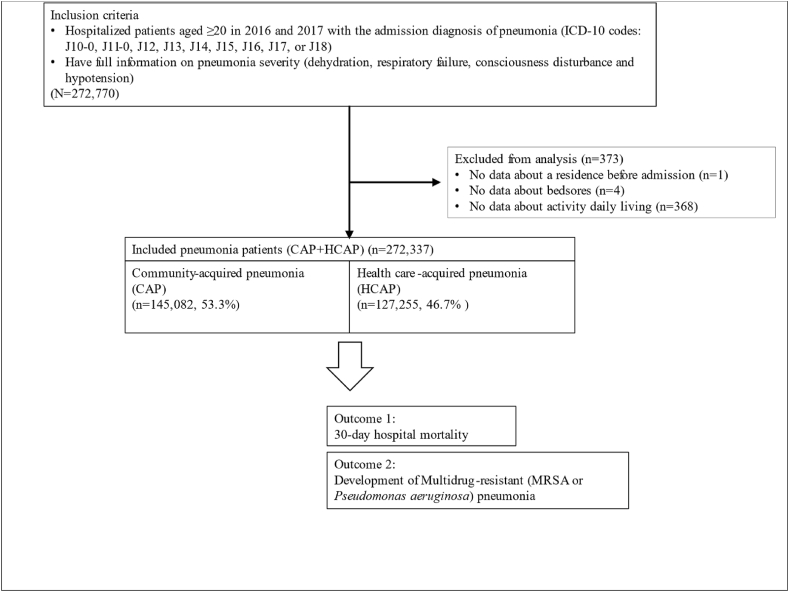
Flowchart outlining the selection and categorization of study participants.

All patients aged ≥20 years who were admitted to hospitals with an admission diagnosis (defined as the disease determined to be the basis for hospitalization and necessary treatment) of pneumonia with available information on disease severity between 2016 and 2017 were identified. The admission diagnosis could by modified later at discharge. Therefore, diseases with infiltration such as idiopathic lung disease, pulmonary embolism, lung cancer or congestive heart failure were excluded in this study. The International Statistical Classification of Diseases, 10th Revision (ICD-10) codes included J10.0 (influenza due to identified influenza virus with pneumonia), J11.0 (influenza due to unidentified influenza virus with pneumonia)J12 (viral pneumonia), J13 (pneumonia due to *Streptococcus pneumonia*), J14 (pneumonia due to *Hemophilus influenza*), J15 (bacterial pneumonia, not elsewhere classified), J16 (pneumonia due to other infectious organisms, not elsewhere classified), J17 (pneumonia in diseases classified elsewhere), and J18 (pneumonia, unspecified organism) [16]. Patients with missing data on variables (n = 373) were excluded from the study (Fig. 1). HCAP was defined as pneumonia in patients with any of the following conditions: hospitalization during the preceding 90 days; residence in a nursing home; or immunosuppressed conditions including cancer, immune disorders, steroid use, immunosuppressant use, and chronic dialysis based on the ATS/IDSA guidelines and previous studies [2,3,5,17]. CAP was defined as pneumonia that did not meet the HCAP criteria.

### 2.3 Outcome measures and clinical assessment

The outcomes of interest were in-hospital mortality within 30-days of admission and development of MDR pneumonia. MRSA (ICD code J15.2) and *P. aeruginosa* (ICD code J15.1) were defined as MDR pathogens based on ATS/IDSA guidelines [2]. Pneumonia due to other pathogens, including *Acinetobacter baumannii* and extended-spectrum beta-lactamase (ESBL)-producing organisms, were not included because they were not identified from the ICD codes and their incidences were considerably smaller than those of MRSA or *P. aeruginosa* in the Japanese population [[18], [19], [20], [21]].

The following patient information was extracted from the database: age, sex, body mass index, smoking status (Brickman index), place of residence before admission (home, nursing home, or extended care facility), non-ambulatory status (yes or no), bedsore (yes or no), dialysis (yes or no), immunosuppression defined as having cancer or immunity disorder or using steroids or immunosuppressants (yes or no), intensive care defined as admission to the intensive care unit, date of admission and discharge, date of the previous admission, diagnoses and comorbidities recorded with ICD-10 codes, and medications.

We also used dehydration (blood urea nitrogen ≥21 mg/mL), respiratory failure (pulse oximetry saturation, SpO2≤90 %), consciousness disturbance, and low blood pressure (systolic blood pressure ≤90 mmHg) included in the A-DROP scoring system [15] proposed by the Japanese Respiratory Society. Concurrently, comorbidities of heart failure, liver failure, and chronic obstructive pulmonary disease (COPD), assessed by the Hugh-Jones grade. The variables were chosen from the database based on a previous study [19,22,23]; however, some were excluded because of multicollinearity.

We compared the baseline characteristics, 30-day hospital mortality, and prevalence of MRSA or *P. aeruginosa* infections, predictors for 30-day hospital mortality, and risk factors for MDR (MRSA or *P. aeruginosa)* infections between patients with CAP and HCAP.

### 2.4 Statistical analysis

Demographic and clinical characteristics are described as frequencies and percentages according to pneumonia classification (all pneumonia [CAP + HCAP], CAP, or HCAP). The difference between CAP and HCAP was compared using chi-square test. Univariable logistic regression was used to test the association between demographic or clinical characteristics and all pneumonia, CAP, or HCAP. Multivariable logistic regression analyses were performed to evaluate the predictors for 30-day hospital mortality and risk factors for MDR (MRSA and *P. aeruginosa)* infection. For the 30-day hospital mortality, we adjusted for age, sex, body mass index, smoking status, residence in a nursing home or extended care facility (yes or no), non-ambulatory status (yes or no), bedsore (yes or no), dialysis (yes or no), immunosuppression (yes or no), intensive care (yes or no), dehydration, respiratory failure, orientation disturbance, low blood pressure (systolic blood pressure ≤90 mmHg), the Hugh-Jones grade for patients with COPD, heart failure, and liver failure. For MDR development, we adjusted for variables associated with patients’ condition before admission (i.e., age, sex, body mass index, smoking status, residence in a nursing home or extended care facility, non-ambulatory status, bedsore, dialysis, immunosuppression, the Hugh-Jones grade, heart failure, and liver failure). These covariates were selected from previous studies [19,23,24], and also selected on the basis of clinical relevance. The results of univariable logistic regression analysis for the covariates are described in Table S1 and Table S2. Before conducting the analysis, we examined the correlations between the variables, removing one of two variables if the correlation was high to avoid multicollinearity. Odds ratios (ORs) and 95 % confidence intervals (CIs) were calculated. Statistical analyses were performed using STATA version 12. All tests were two-tailed, and a P value less than 0.05 was considered statistically significant.

## 3 Results

### 3.1 Patients’ characteristics

A cohort of 272,710 patients was obtained from the database. Individuals with missing data concerning specific variables (n = 373); no available data on residence status before admission [n = 1], bedsores [n = 4], and daily living activity [n = 368]) were excluded from the study. Subsequently, 272,337 patients diagnosed with pneumonia were included in the analysis. Among them, 145,082 (53.3 %) patients were categorized as having CAP, and 127,255 (46.7 %) patients were categorized as having HCAP. The total number of 30-day hospital mortality was 16,119 (5.9 %), and 8638 (3.2 %) developed MDR pneumonia, including 5141 with MRSA (1.9 %) and 3497 with *P. aeruginosa* (1.3 %) infections. There were significant differences between patients with CAP and HCAP for all variables extracted from the database. Patients with HCAP were more likely to be older, female, underweight, and non-ambulatory and have bedsores and comorbidities such as heart failure, liver failure, and advanced COPD than those with CAP. The condition on admission was more severe for HCAP patients than for CAP patients regarding dehydration, respiratory failure, consciousness disturbance, and hypotension. MDR pathogens were more prevalent (4.0 % vs. 2.4 %, P < 0.001) in HCAP patients than in CAP patients, including MRSA (2.4 % vs. 1.4 %, P < 0.001) and *P. aeruginosa* (1.6 % vs. 1.0 %, P < 0.001). The 30-day mortality was significantly higher for HCAP than that for CAP (8.9 % vs. 3.3 %, P < 0.001; Table 1).

**Table 1:** Baseline characteristics of all pneumonia (CAP + HCAP), CAP and HCAP patients.

**Variable**	All cases of pneumonia (N = 272,337)	CAP (N = 145,082)	HCAP(N = 127,255)	p-value
	n, (%)	n, (%)	n, (%)	
Age	80[70–86]	79[68–86]	81 [72–97]	<0.01
20–59	28955 (10.6)	20287 (14.0)	8668 (6.8)	
60–69	34536 (12.7)	19119 (13.2)	15417 (12.1)	
70–79	69997 (25.7)	36801 (25.4)	33196 (26.1)	
80–89	99416 (36.5)	50617 (34.9)	48799 (38.3)	
90 –	39433 (14.5)	18258 (12.6)	21175 (16.6)	
Female sex	160283 (58.9)	83660 (57.7)	76623 (60.2)	
BMI ≤18.5	86167 (31.6)	39245 (27.1)	46922 (36.9)	<0.01
Smoking				
Brinkman Index (– 399)	177573 (65.2)	96627 (66.6)	80946 (63.6)	<0.01
Brinkman Index (400–799)	24161 (8.9)	13251 (9.1)	10910 (8.6)	
Brinkman Index (800–1199)	22998 (8.4)	11547 (8.0)	11451 (9.0)	
Brinkman Index (1200 –)	21525 (7.9)	10354 (7.1)	11171 (8.8)	
missing	26080 (9.6)	13303 (9.2)	12777 (10.0)	
Pneumonia severity				
Dehydration	100869 (37.0)	49127 (33.9)	51742 (40.7)	<0.01
Respiratory failure	99423 (36.5)	45361 (31.3)	54062 (42.5)	<0.01
Consciousness disturbance	34144 (12.5)	14193 (9.8)	19951 (15.7)	<0.01
Hypotension	16212 (6.0)	6726 (4.6)	9486 (7.5)	<0.01
Critical care^a^	7301 (2.7)	2671 (1.8)	4630 (3.6)	<0.01
Non-ambulatory status	77804 (28.6)	30248 (20.8)	47556 (37.4)	<0.01
Bedsore	11654 (4.3)	4270 (2.9)	7384 (5.8)	<0.01
Residence in a nursing home or extended care facility	43475 (16.0)	–	43475 (34.2)	
Hospitalization during the preceding 90 days	47025 (17.3)	–	47025 (37.0)	
Dialysis	4102 (1.5)	–	4102 (3.2)	
Immunosuppression^b^	67594 (24.8)	–	67594 (53.1)	
Comorbidities				
Heart failure	2614 (1.0)	1233 (0.8)	1381 (1.1)	<0.01
Liver failure	4768 (1.8)	2298 (1.6)	2470 (1.9)	<0.01
COPD				<0.01
Hugh Jones I	44710 (16.4)	29895 (20.6)	14815 (11.6)	
Hugh Jones II	41012 (15.1)	25565 (17.6)	15447 (12.1)	
Hugh Jones III	31919 (11.7)	18822 (13.0)	13097 (10.3)	
Hugh Jones IV	49036 (18.0)	26912 (18.5)	22124 (17.4)	
Hugh Jones V	44658 (16.4)	19442 (13.4)	25216 (19.8)	
None	61002 (22.4)	24446 (16.9)	36556 (28.7)	
14 day mortality	10897 (4.0)	3417 (2.4)	7480 (5.9)	
MDR pneumonia	8638 (3.2)	3535 (2.4)	5103 (4.0)	<0.01
MRSA pneumonia	5141 (1.9)	2097 (1.4)	3044 (2.4)	<0.01
Pseudomonas pneumonia	3497 (1.3)	1438 (1.0)	2059 (1.6)	<0.01

Abbreviations: CAP, community acquired pneumonia; HCAP, healthcare-associated pneumonia; BMI, body mass index; COPD, chronic obstructive pulmonary disease; MDR, multidrug-resistant; MRSA, methicillin-resistant Staphylococcus aureus.

^c^P value for difference between CAP and HCAP.

a Critical care: patients who were admitted to ICU, intubated or administered vasopressor on admission.

b Immunosuppressed: patients who have cancer or immunodeficiency, or who use immunosuppressants.

### 3.2 Risk factors for the mortality

In all pneumonia patients, multivariable logistic regression analysis showed that the independent predictors for 30-day mortality were as follows: older age; male sex; being underweight; non-ambulatory status; having a bed sore; residence in a nursing home or extended care facility; hospitalization within 90 days prior to admission; dialysis; immunosuppression; comorbidities such as heart failure, liver failure, and COPD; and pneumonia severity on admission, including dehydration, respiratory failure, consciousness disturbance, and hypotension. These risk factors were shared between CAP and HCAP patients, except for the comorbidity of liver failure (Table 2).

**Table 2:** Risk factors for 30-day in hospital mortality among all pneumonia patients (CAP + HCAP), CAP and HCAP.

Variable	All cases of pneumonia (CAP + HCAP)			CAP			HCAP		
	30-day hospital death (%)	Univariable-adjusted OR	Multivariable-adjusted OR^c^	30-day hospital death (%)	Univariable-adjusted OR	Multivariable-adjusted OR^c^	30-day hospital death (%)	Univariable-adjusted OR	Multivariable-adjusted OR^c^
Age									
20–59	268(0.9)	1	1	76(0.4)	1	1	192(1.7)	1	1
60–69	978(2.8)	2.85(2.43–3.35)	**2.07(1.80**–**2.39)**	193(1.0)	2.31(1.73–3.09)	**1.92(1.46**–**2.54)**	785(5.1)	2.29(1.88–2.78)	**2.02(1.71**–**2.39)**
70–79	3196(4.6)	4.35(3.75–5.03)	**2.65(2.32**–**3.02)**	803(2.2)	4.49(3.48–5.79)	**2.94(2.30**–**3.76)**	2393(7.2)	3.10(2.58–3.71)	**2.45(2.09**–**2.86)**
80–89	7569(7.6)	7.78(6.75–8.98)	**3.59(3.15**–**4.08)**	2285(4.7)	9.99(7.82–12.76)	**4.40(3.46**–**5.58)**	5284(10.8)	4.96(4.16–5.92)	**3.15(2.71**–**3.68)**
90 –	4108(10.4)	11.65(10.07–13.47)	**4.38(3.84**–**5.00)**	1395(8.3)	17.90(13.96–22.95)	**5.83(4.58**–**7.44)**	2713(12.8)	6.44(5.38–7.71)	**3.63(3.10**–**4.25)**
Female sex	5953(5.3)	0.78(0.75–0.81)	**0.71(0.69**–**0.74)**	1820(3.1)	0.77(0.71–0.82)	**0.66(0.62**–**0.71)**	4133(8.2)	0.82(0.78–0.86)	**0.73(0.70**–**0.77)**
BMI ≤18.5	7770(9.0)	1.75(1.68–1.82)	**1.44(1.39**–**1.49)**	2268(6.1)	2.01(1.87–2.15)	**1.63(1.53**–**1.74)**	5502(11.7)	1.46(1.40–1.54)	**1.34(1.28**–**1.40)**
Smoking									
Brinkman Index (– 399)	10,595(6.0)	1	1	3200(3.4)	1	1	7395(9.1)	1	1
Brinkman Index (400–799)	1278(5.3)	0.89(0.83–0.96)	0.97(0.91–1.04)	375(2.9)	0.06(1.04–0.00)	0.98(0.86–1.10)	903(8.3)	0.89(0.82–0.98)	0.97(0.90–1.06)
Brinkman Index (800–1199)	1234(5.4)	0.91(0.85–0.99)	**0.89(0.83**–**0.95)**	306(2.7)	0.89(0.77–1.03)	**0.84(0.74**–**0.96)**	928(8.1)	0.89(0.81–0.98)	**0.90(0.83**–**0.98)**
Brinkman Index (1200 –)	1207(5.6)	0.84(0.77–0.90)	**0.87(0.81**–**0.93)**	314(3.1)	0.83(0.72–0.96)	**0.86(0.75**–**0.98)**	893(8.0)	0.79(0.72–0.87)	**0.87(0.80**–**0.94)**
Non ambulatory status	10,452(13.4)	4.62(4.43–4.82)	**1.79(1.72**–**1.87)**	3115(11.5)	6.10(5.66–6.58)	**2.13(1.98**–**2.28)**	7337(15.4)	3.40(3.23–3.58)	**1.61(1.53**–**1.69)**
Bedsore	2047(17.6)	2.87(2.70–3.05)	**1.43(1.35**–**1.51)**	612(16.7)	3.74(3.35–4.16)	**1.46(1.31**–**1.61)**	1435(19.4)	2.26(2.10–2.43)	**1.40(1.30**–**1.49)**
Dialysis	310(7.6)	1.51(1.30–1.74)	**1.38(1.21**–**1.57)**	–		–	310(7.6)	0.99(0.86–1.14)	**1.31(1.15**–**1.49)**
Immunosuppression^b^	6130(9.6)	2.00(1.92–2.09)	**2.28(2.19**–**2.37)**	–		–	6130(9.6)	1.26(1.20–1.32)	**2.14(2.03**–**2.26)**
Residence in a nursing home or extended care facility	5039(11.6)	2.02(1.93–2.11)	**1.09(1.04**–**1.13)**	–		–	5039(11.6)	1.43(1.36–1.51)	**1.15(1.09**–**1.21)**
Hospitalization during the preceding 90 days	4153(8.8)	1.56(1.49–1.63)	**1.43(1.37**–**1.49)**	–		–	4153(8.8)	0.99(0.94–1.04)	**1.39(1.33**–**1.46)**
Pneumonia severity									
Dehydration	10,720(10.6)	3.25(3.11–3.39)	**1.77(1.71**–**1.84)**	3335(7.3)	3.90(3.61–4.22)^c^	**1.94(1.81**–**2.08)**	7385(14.3)	2.87(2.73–3.02)	**1.70(1.62**–**1.77)**
Respiratory failure	11,271(11.3)	4.55(4.35–4.75)	**2.00(1.92**–**2.08)**	3302(7.9)	5.13(4.75–5.54)	**2.15(2.01**–**2.31)**	,969(14.7)	3.88(3.68–4.09)	**1.92(1.83**–**2.01)**
Consciousness disturbance	6506(19.1)	5.29(5.08–5.50)	**1.78(1.71**–**1.85)**	2005(16.5)	6.80(6.33–7.30)	**1.88(1.75**–**2.01)**	4501(22.6)	4.27(4.06–4.48)	**1.73(1.65**–**1.81)**
Hypotension	3236(20.0)	4.95(4.72–5.20)	**1.96(1.87**–**2.06)**	945(16.3)	6.06(5.56–6.61)	**2.11(1.92**–**2.30)**	2291(24.2)	4.08(3.85–4.33)	**1.89(1.78**–**2.01)**
Critical care	1773(24.3)	6.58(6.17–7.01)	**1.82(1.71**–**1.94)**	587(28.2)	10.48(9.39–11.68)	**2.73(2.43**–**3.06)**	1186(25.6)	4.64(4.29–5.02)	**1.53(1.41**–**1.65)**
Comorbidities									
Heart failure	428(16.4)	2.44(2.15–2.77)	**1.62(1.44**–**1.82)**	139(12.7)	2.39(1.92–2.98)	**1.52(1.25**–**1.86)**	289(20.9)	2.41(2.06–2.80)	**1.65(1.43**–**1.91)**
Liver failure	297(6.2)	1.18(1.02–1.36)	**1.22(1.07**–**1.39)**	75(3.4)	1.07(0.81–1.43)	1.25(0.97–1.61)	222(9.0)	1.15(0.97–1.36)	**1.21(1.04**–**1.41)**
COPD									
Hugh Jones I	346(0.8)	1	1	118(0.4)	1	1	228(1.5)	1	1
Hugh Jones II	504(1.2)	1.34(1.12–1.59)^c^	**1.16(1.01**–**1.34)**	162(0.6)	1.37(1.03–1.82)	1.11(0.88–1.42)	342(2.2)	1.24(0.99–1.54)	1.17(0.99–1.39)
Hugh Jones III	652(2.0)	2.13(1.81–2.52)^c^	**1.59(1.39**–**1.82)**	161(0.9)	1.59(1.19–2.12)	1.18(0.92–1.50)	491(3.8)	2.20(1.79–2.70)	**1.74(1.48**–**2.05)**
Hugh Jones IV	1618(3.3)	3.32(2.87–3.85)^c^	**2.00(1.78**–**2.26)**	484(1.8)	3.16(2.48–4.03)	**1.85(1.51**–**2.28)**	1134(5.1)	2.93(2.43–3.53)	**1.99(1.72**–**2.31)**
Hugh Jones V	6043(13.5)	15.96(13.91–18.31)	**4.97(4.44**–**5.57)**	1775(10.0)	17.17(13.71–21.49)	**5.10(4.19**–**6.20)**	4268(16.9)	11.95(10.05–14.22)	**4.65(4.04**–**5.35)**

Abbreviations: CAP, community acquired pneumonia; HCAP, healthcare-associated pneumonia; BMI, body mass index; COPD, chronic obstructive pulmonary disease; MRSA, methicillin-resistant Staphylococcus aureus.

All cases of pneumonia, n = 272,337 (16,119 with 30-day hospital mortality and 256,218,699 without 30-day hospital mortality); CAP, n = 145,082 (4752 with 30-day hospital mortality and 140,330 without 30-day hospital mortality); HCAP, n = 127,255 (11,367 with 30-day hospital mortality and 114,888 without 30-day hospital mortality).

^a^Critical care: patients who were admitted to ICU, intubated or administered vasopressor on admission.

b Immunosuppression: patients who have cancer or immunodeficiency, or who use immunosuppressants.

c Adjusted for all variables listed in this table.

### 3.3 Risk factors for the MRSA and *P. aeruginosa* infection

Table 3 shows the risk factors for pneumonia caused by MDR. Underweight, non-ambulatory status, bedsores, and COPD were common dependent risk factors for MDR pneumonia. Table 4 and Table 5 show the risk factors for pneumonia due to MRSA and *P. aeruginosa* infection separately. Underweight, prior hospitalization, and COPD were associated with both MRSA and *P. aeruginosa* pneumonia development. Age (>80 years old), male, non-ambulatory status, bedsores, residence in a nursing home or extended care facility, dialysis, and immunosuppression were significantly associated with MRSA pneumonia development but not with *Pseudomonas* pneumonia. When we compared CAP and HCAP, five out of six risk factors for MRSA pneumonia (male, underweight, non-ambulatory status, bedsore, and COPD), and three out of three risk factors for *P. aeruginosa* infection (female, underweight, COPD) were identical between CAP and HCAP patients.

**Table 3:** Risk factors for pneumonia due to multidrug-resistant pathogens (MRSA and P. Aeruginosa) in all pneumonia (CAP + HCAP), CAP, and HCAP patients.

Variable	All cases of pneumonia (CAP + HCAP)			CAP			HCAP		
	Incidence(%)	Univariable-adjusted OR	Multivariable-adjusted OR^b^	Incidence(%)	Univariable-adjusted OR	Multivariable-adjusted OR^b^	Incidence(%)	Univariable-adjusted OR	multivariable-adjusted OR^b^
Age >80 years	3.3	1.09(1.04–1.14)	**0.86(0.82**–**0.90)**	2.7	1.19(1.12–1.28)	0.94(0.88–1.01)	3.9	0.95(0.90–1.01)	**0.80(0.75**–**0.85)**
Female sex	3.2	1.03(0.99–1.08)	0.97(0.93–1.02)	2.6	1.09(1.01–1.16)	1.01(0.92–1.07)	4.1	1.03(0.97–1.09)	0.96(0.90–1.02)
BMI ≤18.5	4.5	1.80(1.72–1.88)	**1.58(1.51**–**1.66)**	3.6	1.79(1.67–1.92)	**1.69(1.56**–**1.81)**	5.3	1.68(1.58–1.77)	**1.50(1.42**–**1.59)**
Smoking status									
Brinkman Index (– 399)	3.3	1.0	1	2.6	1.0	1	4.2	1.0	1
Brinkman Index (400–799)	2.9	0.86(0.79–0.93)	**0.89(0.82**–**0.97)**	2.3	0.92(0.81–1.04)	0.92(0.81–1.05)	3.6	0.82(0.73–0.91)	**0.87(0.78**–**0.97)**
Brinkman Index (800–1199)	3.0	0.84(0.77–0.92)	**0.88(0.81**–**0.96)**	2.1	0.79(0.69–0.91)	**0.84(0.73**–**0.96)**	3.8	0.85(0.76–0.94)	0.92(0.82–1.02)
Brinkman Index (1200 –)	3.0	0.86(0.79–0.94)	**0.90(0.82**–**0.98)**	2.1	0.76(0.66–0.88)	**0.80(0.69**–**0.93)**	3.9	0.88(0.79–0.98)	0.95(0.86–1.06)
Non ambulatory status	4.2	1.56(1.49–1.64)	**1.16(1.10**–**1.23)**	3.4	1.50(1.39–1.62)	**1.22(1.12**–**1.33)**	4.8	1.42(1.34–1.51)	**1.12(1.04**–**1.20)**
Bedsore	5.1	1.64(1.50–1.79)	**1.20(1.09**–**1.31)**	4.1	1.63(1.39–1.90)	**1.21(1.03**–**1.43)**	5.7	1.50(1.35–1.66)	**1.19(1.07**–**1.32)**
Residence in a nursing home or extended care facility	4.5	1.56(1.48–1.65)	**1.20(1.13**–**1.27)**	–	–	–	4.5	1.27(1.19–1.35)	**1.11(1.03**–**1.21)**
Hospitalization during the preceding 90 days	5.0	1.83(1.74–1.92)	**1.66(1.58**–**1.74)**	–	–	–	5.0	1.50(1.42–1.59)	**1.50(1.40**–**1.60)**
Dialysis	4.4	1.41(1.21–1.64)	**1.31(1.12**–**1.52)**	–	–	–	4.4	1.09(0.93–1.27)	1.16(0.99–1.36)
Immunosuppression^a^	3.4	1.08(1.02–1.13)	**1.06(1.01**–**1.12)**	–	–	–	3.4	0.69(0.65–0.73)	**0.92(0.86**–**0.99)**
Comorbidities									
Heart failure	3.9	1.18(0.97–1.45)	1.03(0.84–1.26)	3.1	1.18(0.85–1.64)	1.01(0.73–1.40)	4.6	1.15(0.89–1.49)	1.03(0.80–1.33)
Liver failure	3.2	1.00(0.85–1.17)	1.01(0.86–1.19)	2.8	1.13(0.88–1.46)	1.17(0.91–1.51)	3.6	0.87(0.70–1.08)	0.91(0.73–1.13)
COPD									
Hugh Jones I	1.7	1.0	1	1.4	1.0	1	2.4	1.0	1
Hugh Jones II	2.3	1.33(1.21–1.47)	**1.35(1.23**–**1.48)**	1.9	1.33(1.16–1.51)	**1.39(1.22**–**1.58)**	3.0	1.28(1.11–1.47)	**1.27(1.11**–**1.46)**
Hugh Jones III	2.9	1.65(1.50–1.82)	**1.65(1.50**–**1.82)**	2.3	1.57(1.37–1.80)	**1.66(1.45**–**1.90)**	3.8	1.61(1.40–1.85)	**1.58(1.37**–**1.81)**
Hugh Jones IV	3.3	1.88(1.72–2.05)	**1.81(1.66**–**1.98)**	2.8	1.87(1.65–2.12)	**1.96(1.73**–**2.21)**	3.9	1.69(1.49–1.92)	**1.61(1.42**–**1.83)**
Hugh Jones V	4.3	2.49(2.28–2.71)	**2.15(1.97**–**2.35)**	3.5	2.36(2.08–2.68)	**2.28(2.01**–**2.60)**	4.9	2.18(1.93–2.46)	**1.95(1.72**–**2.20)**

Abbreviations: CAP, community-acquired pneumonia; HCAP, healthcare-associated pneumonia; BMI, body mass index; COPD, chronic obstructive pulmonary disease; MRSA, methicillin-resistant Staphylococcus aureus; MDR, multidrug-resistant.

All cases of pneumonia, n = 272,337 (8638 with MDR and 263,699 without MDR); CAP, n = 145,082 (3535 with MDR and 141,547 without MDR); HCAP, n = 127,255 (5103 with MDR and 122,152 without MDR).

a Immunosuppression: patients who have cancer or immunodeficiency, or who use immunosuppressants.

b Adjusted for all variables listed in this table.

**Table 4:** Risk factors for pneumonia due to MRSA in all pneumonia (CAP + HCAP), CAP, and HCAP patients.

Variable	All cases of pneumonia (CAP + HCAP)		CAP		HCAP	
	Incidence(%)	Multivariable-adjusted OR^b^	Incidence(%)	Multivariable-adjusted OR^b^	Incidence(%)	multivariable-adjusted OR^b^
Age >80 years	2.2	**1.12(1.05**–**1.19)**	1.8	**1.24 (1.13–1.36)**	3.9	1.03 (0.95–1.11)
Female sex	1.8	**0.85(0.80**–**0.90)**	1.4	**0.86 (0.78–0.94)**	2.4	**0.84 (0.77–0.91)**
BMI ≤18.5	2.5	**1.29(1.22**–**1.36)**	1.9	**1.31 (1.19–1.44)**	3.0	**1.27 (1.18–1.37)**
Smoking status						
Brinkman Index (– 399)	2.0	1	1.5	1	4.2	1
Brinkman Index (400–799)	1.8	0.97(0.87–1.08)	1.5	1.04 (0.89–1.21)	2.6	0.92 (0.80–1.06)
Brinkman Index (800–1199)	1.8	0.92(0.82–1.02)	1.3	0.83 (0.69–0.99)	2.1	0.98 (0.85–1.12)
Brinkman Index (1200 –)	1.8	0.91(0.82–1.02)	1.2	0.79 (0.65–0.95)	2.3	0.99 (0.86–1.14)
Non ambulatory status	3.0	**1.38(1.29**–**1.48)**	2.5	**1.56 (1.40–1.73)**	2.3	**1.27 (1.16–1.38)**
Bedsore	3.7	**1.34(1.21**–**1.48)**	2.9	**1.29 (1.07–1.56)**	2.3	**1.36 (1.20–1.53)**
Residence in a nursing home or extended care facility	3.2	**1.34(1.25**–**1.44)**	–	–	3.2	**1.41 (1.27–1.56)**
Hospitalization during the preceding 90 days	2.6	**1.31(1.23**–**1.40)**	–	–	2.6	**1.30 (1.19–1.41)**
Dialysis	3.4	**1.88(1.58**–**2.24)**	–	–	3.4	**1.82 (1.52–2.19)**
Immunosuppression^a^	2.0	**1.13(1.06**–**1.21)**	–	–	2.0	**1.10 (1.01–1.21)**
Comorbidities						
Heart failure	2.6	1.06(0.83–1.35)	2.2	1.09 (0.74–1.61)	2.9	1.03 (0.75–1.42)
Liver failure	1.8	1.02(0.82–1.26)	1.4	1.01 (0.71–1.43)	2.2	1.02 (0.78–1.34)
COPD						
Hugh Jones I	0.9	1	0.8	1	1.2	1
Hugh Jones II	1.2	**1.21(1.06**–**1.38)**	1.0	**1.19 (1.00–1.43)**	1.5	1.21 (0.99–1.48)
Hugh Jones III	1.5	**1.47(1.29**–**1.68)**	1.2	**1.45 (1.21–1.74)**	1.8	**1.46 (1.20–1.78)**
Hugh Jones IV	1.8	**1.73(1.54**–**1.95)**	1.5	**1.68 (1.43–1.98)**	2.2	**1.72 (1.44–2.06)**
Hugh Jones V	2.8	**2.26(2.01**–**2.55)**	2.4	**2.29 (1.94–2.71)**	3.1	**2.19 (1.84–2.59)**

Abbreviations: CAP, community-acquired pneumonia; HCAP, healthcare-associated pneumonia; BMI, body mass index; COPD, chronic obstructive pulmonary disease; MRSA, methicillin-resistant Staphylococcus aureus; MDR, multidrug-resistant.

All cases of pneumonia, n = 272,337 (5141 with MRSA and 267,196 without MRSA); CAP, n = 145,082 (2097 with MRSA and 142,985 without MRSA); HCAP, n = 127,255 (3044 with MRSA and 124,211 without MRSA).

a Immunosuppression: patients who have cancer or immunodeficiency, or who use immunosuppressants.

b Adjusted for all variables listed in this table.

**Table 5:** Risk factors for pneumonia due to P. Aeruginosa in all pneumonia (CAP + HCAP), CAP, and HCAP patients.

Variable	All cases of pneumonia (CAP + HCAP)		CAP		HCAP	
	Incidence(%)	Multivariable-adjusted OR^b^	Incidence(%)	Multivariable-adjusted OR^b^	Incidence(%)	multivariable-adjusted OR^b^
Age >80 years	1.1	**0.60(0.56**–**0.65)**	0.9	**0.64 (0.58–0.72)**	1.3	**0.57 (0.52–0.62)**
Female sex	1.4	**1.18(1.10**–**1.27)**	1.2	**1.20 (1.07–1.35)**	1.7	**1.16 (1.05–1.28)**
BMI ≤18.5	2.0	**2.09(1.95**–**2.23)**	1.7	**2.36 (2.12–2.62)**	2.3	**1.89 (1.72–2.06)**
Smoking status						
Brinkman Index (– 399)	1.3	1	1.1	1	1.7	1
Brinkman Index (400–799)	1.1	**0.79(0.70**–**0.91)**	0.8	0.76 (0.62–0.94)	1.4	0.82 (0.69–0.97)
Brinkman Index (800–1199)	1.2	**0.85(0.75**–**0.97)**	0.9	0.86 (0.69–1.06)	1.5	0.85 (0.72–1.00)
Brinkman Index (1200 –)	1.2	0.89(0.78–1.02)	0.8	0.84 (0.66–1.05)	1.6	0.91 (0.77–1.08)
Non ambulatory status	1.3	**0.87(0.80**–**0.95)**	0.9	**0.78 (0.68–0.91)**	1.5	0.92 (0.82–1.03)
Bedsore	1.4	0.93(0.79–1.10)	1.2	1.07 (0.80–1.44)	1.5	0.88 (0.72–1.08)
Residence in a nursing home or extended care facility	1.3	0.97(0.87–1.07)	–	–	1.3	0.78 (0.68–0.89)
Hospitalization during the preceding 90 days	2.4	2.22(2.06–2.39)	–	–	2.5	1.76 (1.58–1.96)
Dialysis	1.0	0.64(0.47–0.87)	–	–	1.0	0.52 (0.38–0.71)
Immunosuppression^a^	1.3	0.95(0.87–1.03)	–	–	1.4	0.74 (0.66–0.82)
Comorbidities						
Heart failure	1.3	0.98(0.69–1.38)	0.9	1.01(0.73–1.40)	1.7	1.04 (0.68–1.58)
Liver failure	1.4	1.00(0.78–1.28)	1.4	1.17(0.91–1.51)	1.3	0.77 (0.54–1.09)
COPD						
Hugh Jones I	0.8	1	0.6	1	1.3	1
Hugh Jones II	1.2	1.51(1.32–1.73)	1.0	**1.66 (1.36–2.01)**	1.6	**1.34 (1.10–1.62)**
Hugh Jones III	1.5	1.86(1.62–2.13)	1.1	**1.95 (1.59–2.39)**	2.0	**1.68 (1.39–2.04)**
Hugh Jones IV	1.5	1.91(1.68–2.17)	1.3	**2.35 (1.95–2.82)**	1.7	**1.50 (1.26–1.80)**
Hugh Jones V	1.5	1.94(1.70–2.22)	1.2	**2.16 (1.76–2.65)**	1.8	**1.67 (1.39–2.00)**

Abbreviations: CAP, community acquired pneumonia; HCAP, healthcare-associated pneumonia; BMI, body mass index; COPD, chronic obstructive pulmonary disease; MDR, multidrug-resistant.

All cases of pneumonia, n = 272,337 (3497 with Pseudomonas pneumonia and 268,840 without Pseudomonas pneumonia); CAP, n = 145,081(1438 with Pseudomonas pneumonia and 143,643 without Pseudomonas pneumonia); HCAP, n = 127,256 (2059 with Pseudomonas pneumonia and 125,197 without Pseudomonas pneumonia).

a Immunosuppression: patients who have cancer or immunodeficiency, or who use immunosuppressants.

b Adjusted for all variables listed in this table.

## 4 Discussion

This study identified the individual risk factors for 30-day mortality and infection with MDR pathogens, including MRSA and *P. aeruginosa,* in pneumonia patients who resided in the community using a national discharge database of acute hospitals in Japan. By classifying patients into CAP and HCAP groups, we found that patients with HCAP were associated with significantly higher mortality and infection with MDR pathogens than patients with CAP, but they shared almost the same risk factors for 30-day hospital mortality and the development of MRSA and *P. aeruginosa* infection. In addition, the risk factors for MRSA infection were different from those for *P. aeruginosa* infection. This study is the first to comprehensively assess the risk factors for pneumonia by studying the largest number of pneumonia patients from the community compared to previous studies.

We found that patients’ background characteristics such as age; male sex; being underweight; residence in a nursing home or extended care facility; being bedridden; prior admission; immunosuppression; and comorbidities of heart failure, liver failure, and COPD, as well as severity of conditions at admission, including dehydration, respiratory failure, conscious disturbance, and hypotension were significantly associated with 30-day hospital mortality. These factors have been consistently reported in older studies in the late 1990s and in more recent studies [[25], [26], [27], [28], [29]]. Bedsores were further identified as an independent risk factor for mortality. Predisposing factors for pressure ulcers include immobility, malnutrition, reduced skin perfusion, and sensory loss [30], and these factors overlap with the known risk factors for pneumonia mortality [31,32]. After classifying pneumonia patients into CAP and HCAP groups, we found that the risk factors for 30-day mortality were similar between them, except for liver failure.

The 2005 ATS/ADSA guidelines defined the following factors to identify HCAP patients who may have been exposed to healthcare and thus, were at risk of carrying MDR pathogens: patients with hospitalization within 90 days prior to admission; nursing home residents; patients receiving home infusion therapy, hemodialysis and/or wound care; and patients with family members with MDR pathogens [3]. However, later studies have reported that the weight of individual risk factors may not be equal [27] and can lead to misuse of antibiotics. Our study identified that hospitalization within 90 days prior to admission (OR 1.66, 95 % CI 1.58–1.74), residing in a nursing home (OR 1.20, 95 % CI 1.13–1.27), hemodialysis (OR 1.31, 95 % CI 1.12–1.52), and immunosuppression (OR 1.06, 95 % CI 1.01–1.12) were significantly associated with the development of MDR pneumonia. While these are independent risk factors for MDR pneumonia, the estimated ORs vary widely. Hence, treating all HCAP patients equally with broad-spectrum antimicrobials may lead to overtreatment of patients with low OR for MDR risk factors.

In addition to the MDR pneumonia risk factors included in the HCAP definition, we identified that being underweight and having a non-ambulatory status, bedsores, and COPD were also independent risk factors for the development of MDR pneumonia. Non-ambulatory status and COPD have also been shown to be risk factors in previous studies [33]. In our study, COPD grade showed a clear dose-response association with MDR pneumonia development; therefore, the association may be robust [34,35]. Being underweight and having bedsores are indications of malnutrition in patients, making them prone to infection with MDR pathogens. These factors were similar in patients with CAP and HCAP and could be added to the individual risk factors to predict MDR pneumonia.

Although the incidence of MDR pneumonia was significantly higher in HCAP patients than in CAP patients (4.0 % vs. 2.4 %), the incidence was not high compared with other types of pneumonia, such as HAP or Ventilator-Associated Pneumonia [24]. If the 2005 ATS/ADSA guidelines are followed and broad-spectrum antimicrobial agents are equally used in all HCAP patients, overtreatment would be likely in the majority of patients with HCAP. Therefore, it is important to assess patients’ individual risk factors for MDR judiciously to reserve the use of broad-spectrum antimicrobial agents. The ATS/IDSA, consequently removed the concept of HCAP from their new guidelines [12,13].

Previous cohort studies evaluating the predictors of MDR pneumonia have combined MRSA and *P. aeruginosa* [19,21,27]. However, it is important to identify the predictors for MRSA and *P. aeruginosa* infection separately to avoid overuse of broad-spectrum antibiotics because of differences in their antibacterial spectrum. This study revealed that the risk factors for MRSA and *P. aeruginosa* infections were not identical. We discerned that dialysis (OR 1.88, 95%CI, 1.58–2.24) for MRSA pneumonia, and low body weight (OR 2.09, 95%CI, 1.95–2.23) for *P. aeruginosa* pneumonia constitute distinctive risks. Clinicians are encouraged to ascertain the pathogen of the infection as comprehensively as possible utilizing this evidence, whilst referring to locally validated risks as suggested by the 2019 guidelines for CAP [13]. Furthermore, an exploration of pathogenesis concerning the rationale behind the differences between MRSA and *P. aeruginosa* is warranted.

This study had several limitations. First, the DPC database lacks comprehensive details in areas such as socioeconomic status, laboratory tests, radiographic images, and microbiological tests. Based on these data, it can be deduced that clinicians had comprehensibly confirmed the diagnosis; however, the specific criteria they used are not clear. This extends to how they differentiated between colonization and infection in patients with MDR infections. Second, while this study includes all patients from acute hospitals using DPC, its findings may not apply universally as all participants were Japanese. Third, patients were selected based on specific ICD-10 codes (J10 to J18). Previous studies showed that these codes reliably identify pneumonia [16], but this method may have excluded some pneumonia patients with other codes, and included some misdiagnosed patients. Lastly, the study uses data collected between 2016 and 2017, which, while not the most current, we believe is still significant. We contend that the risks for 30-day mortality and MDR infection remain fairly consistent over time.

## 5 Conclusions

This observational study from a nationwide discharge database of acute care hospitals described the clinical characteristics and frequency of MDR infections including MRSA and *P. aeruginosa,* and their outcomes in patients with CAP and HCAP. The risk factors for MDR infection, including MRSA and *P. aeruginosa,* and mortality were nearly identical in patients with CAP and HCAP. The risk factors for MRSA and *P. aeruginosa* infections were not identical. The assessment of individual risk factors for mortality and MDR infection, and including HCAP in CAP could contribute to the improvement and simplification of empiric therapy. This may also be useful to assess predictors for MRSA and *P. aeruginosa* infections separately to minimize the overuse of broad-spectrum antibiotics.

## Financial contribution

This research was supported by the 10.13039/100004423World Health Organization Center for Health Development (10.13039/100004423WHO Kobe Center, WKC: K18003).

## Data availability

The data is not deposited into a publicly available repository.

## CRediT authorship contribution statement

**Tomohiko Ukai:** Writing – review & editing, Writing – original draft, Software, Methodology, Formal analysis, Conceptualization. **Takaya Maruyama:** Writing – review & editing, Writing – original draft, Supervision, Methodology. **Shinichi Tomioka:** Funding acquisition, Formal analysis. **Takumi Fukui:** Writing – original draft, Formal analysis, Data curation. **Shinya Matsuda:** Writing – review & editing, Supervision, Project administration, Investigation, Data curation. **Kiyohide Fushimi:** Writing – review & editing, Supervision, Project administration, Data curation. **Hiroyasu Iso:** Writing – review & editing, Supervision.

## Declaration of competing interest

The authors declare that they have no known competing financial interests or personal relationships that could have appeared to influence the work reported in this paper.
