# Supplementary material for: Predictors of hospital mortality and multidrug-resistant pathogens in hospitalized pneumonia patients residing in the community
Source: Heliyon. 2023 Nov 15;9(12):e22303. doi: 10.1016/j.heliyon.2023.e22303 (PMC10730438; doi:10.1016/j.heliyon.2023.e22303)
Supplement: Multimedia component 1 [file mmc1.docx]

ONLINE SUPPLEMENT

**Predictors of hospital mortality and multidrug-resistant pathogens**

**in hospitalized pneumonia patients resided in community**

Tomohiko Ukai^1,2^, Takaya Maruyama^3^, Shinichi Tomioka^4^, Takumi Fukui^4^, Shinya Matsuda^4^, Kiyohide Fushimi^5^, Michael S. Niederman^6*^, Hiroyasu Iso^1^.

^1^ Public Health, Department of Social Medicine, Osaka University Graduate School of Medicine, 2-2 Yamadaoka, Suita, Osaka 565-0871, Japan.

^2^ Devision of Public Health, Osaka Institute of Public Health, 1-3-69 Nakamichi, Higashinari-ku, Osaka, Osaka 537-0025, Japan.

^3^ Department of Respiratory Medicine, National Hospital Organization Mie National Hospital, 357 Ohzatokubota, Tsu, Mie 514-0125, Japan

^4^ Department of Preventive Medicine and Community Health, School of Medicine, University of Occupational and Environmental Health, 1-1, Iseigaoka, Yahatanishiku, Kitakyushu, 807-8555, Japan

^5^ Department of Health Policy and Informatics, Tokyo Medical and Dental University, 1-5-45, Yushima, Bunkyoku, Tokyo, 113-8510, Japan.

^6^ Division of Pulmonary and Critical Care Medicine, New York Presbyterian/Weill Cornell Medical Center, 425 East 61(st) Street, New York, NY 10065, USA.

(*)Correspondence: Dr Michael S. Niederman, New York Presbyterian Hospital, Weill Cornell Medical Center, 425 E 61st St, New York, NY 10065; e-mail: [msn9004@med.cornell.edu](mailto:msn9004@med.cornell.edu)

The Diagnosis Procedure Combination (DPC) is a case-mix patient classification system originally developed in Japan in 2012. This system is linked with a lump-sum system for inpatients in acute care hospitals, so called the DPC per-diem payment system (DPC/ PDPS). All the 82 academic hospitals (80 university hospitals, National Cancer Center and Nation Cerebral and Cardiovascular Center) are obliged to adopt the DPC system, but adoption by community hospitals is voluntary. Key objectives of the DPC system are to implement a standardized electronic claims system and to provide transparency of hospital performance. The DPC data are used to identify, track, and analyze national trends in health care utilization, access, quality, outcomes, and costs. Furthermore, the DPC database can be utilized for clinical epidemiology and health services research because it has several clinical data as well as detailed process data.

The structure of DPC.

In the DPC case-mix algorithm, diagnosis, procedure, and comorbidities / complications are key elements for classification. The DPC codes are structured with the following components: (i) 18 Major Diagnosis Categories and 2,927 diagnostic groups, as of 2014 (ii) type of admission, (iii) patient backgrounds (age, Japan Coma Scale, birth weight for neonates, etc.), (iv) surgical procedures, (v) adjuvant therapies, and (vi) comorbidities / complications. Diagnoses, comorbidities and complications are coded using the International Classification of Diseases, 10th Revision codes (ICD-10). Examinations, procedures and pharmaceuticals are coded in the Japanese original codes, as defined in the fee schedule of the national health insurance system.

The DPC database

The MHLW electronically collects the DPC data for the purpose of health policy planning including the refinement of case-mix classification and the revision of DPC-based fee schedule. The DPC Study Group, a government-funded academic group, also collects the copies of the DPC electronic data independently of the MHLW for research purpose. The data collection period was July 1 to October 31 during 2002-2005, and July 1 to December 31 during 2006 to 2010. Since 2011, data has been collected through the year (January 1 to December 31).

All the data for each patient are recorded at discharge. To optimize the accuracy of the recorded diagnoses, physicians in charge are obliged to record the diagnoses with reference to medical charts. Licensed medical information managers and trained medical clerks accurately record the dates of all major and mi- nor procedures and of drugs and devices use. Because the entry of accurate data is mandatory to obtain the DPC-based reimbursement of medical fee, hospitals have a strong incentive for data compliance. Hospitals send all the anonymized data to the MHLW. Copies of all the data are also sent to the DPC Study Group, and data are compiled in the database server in the Department of Clinical Epidemiology and Health Economics, School of Public Health, The University of Tokyo.

Diagnoses are recorded with text data in Japanese language and ICD-10 codes. Up to 12 diagnoses for each admission can recorded. One diagnosis each is coded for “main diagnosis”, “admission-precipitating diagnosis”, “most resource-consuming diagnosis” and “second most resource-consuming diagnosis” and maximum of four diagnoses each is coded for “comorbidities present on admission” and “complications arising after admission”. Report of “main diagnosis”, “admission-precipitating diagnosis” and “most resource-consuming diagnosis” are mandatory, whereas recording of “second most resource-consuming diagnosis”, “comorbidities present on admission” and “complications arising after admission” are voluntary. Using Quan’s protocol, each ICD-10 code of comorbidity is converted into a score, and is summed up for each patient to calculate a Charlson comorbidity index.

Hospital and patient data include: unique identifiers of the hospitals; location of the hospitals; zip codes of patients’ residing area; type of admission (urgent or elective); type of psychiatric admission (voluntary or involuntary); ambulance service use; patients’ age and sex; smoking index (pack years); pregnancy; and dis- position of patient (discharged to home, discharged to a nursing facility, discharged to other hospital, or died in hospital).

Procedure data include: anesthesia, surgery, rehabilitation and other procedures (including tracheal intubation, mechanical ventilation, blood purification, etc.) coded with Japanese original codes; duration of anesthesia (min); volume of blood transfusion (ml); and independent codes for pharmaceuticals and devices used. The dates of procedures, the dates of using drugs and devices, the dates of admission to and discharge from hospitals, and admission to and dis- charge from special care units (including intensive care unit, coronary care unit, stroke care unit, or neonatal intensive care unit) are all recorded, and thus an interval between the start and the end of any process can be calculated (e.g. duration of mechanical ventilation, duration of chest tube drainage, total length of stay, postoperative length of stay, and length of stay in intensive care unit). Clinical data include (i) body weight and height; (ii) Japan Coma Scale at admission and discharge; (iii) TNM Classification and Stage for primarily-diagnosed cancer; (iv) modified Rankin scale at admission and discharge for patients with neurological diseases; (v) Hugh–Jones classifications for patients with lung diseases; (vi) New York Heart Association classification for patients with heart diseases; (vii) Canadian Cardiovascular Society classification for patients with heart diseases; (vii) Canadian Cardiovascular Society classification for patients with angina pectoris; (viii) Killip classification for patients with acute myocardial infarction; (ix) A-DROP scoring system for pneumonia patients; (x)Child–Pugh classification for patients with liver cirrhosis22); (xi) Japanese severity classification for patients with acute pancreatitis; (xii) the date of stroke onset; (xiii) Burn Index; (xiv); Activity of Daily Living scores at admission and discharge, which can be converted into Barthel Index; and (xv) Global Assessment of Functioning Scale at admission and duration of mechanical restraint for psychiatric patients. The database also includes estimated total costs based on reference prices in the Japanese national fee schedule that determine item-by-item prices for surgical, pharmaceutical, laboratory, and other inpatient services.
