# Supplementary material for: Predictors of hospital mortality and multidrug-resistant pathogens in hospitalized pneumonia patients residing in the community
Source: Heliyon. 2023 Nov 15;9(12):e22303. doi: 10.1016/j.heliyon.2023.e22303 (PMC10730438; doi:10.1016/j.heliyon.2023.e22303)
Supplement: Multimedia component 2 [file mmc2.docx]

| Table S1. Results of univariable logistic analysis for 30-day in-hospital mortality for individual potential risk factors. | | | | | | |  |
| --- | --- | --- | --- | --- | --- | --- | --- |
|  | All cases of pneumonia (CAP+HCAP) |  | CAP |  | HCAP |  | |
| Variable | Univariable-adjusted OR | P-value | Univariable-adjusted OR | P-value | Univariable-adjusted OR | P-value | |
| Age |  |  |  |  |  |  | |
| 20 – 59 | 1 |  | 1 |  | 1 | 1 | |
| 60 – 69 | 2.85(2.43-3.35) | <0.001 | 2.31(1.73-3.09) | <0.001 | 2.29(1.88-2.78) | <0.001 | |
| 70 – 79 | 4.35(3.75-5.03) | <0.001 | 4.49(3.48-5.79) | <0.001 | 3.10(2.58-3.71) | <0.001 | |
| 80 – 89 | 7.78(6.75-8.98) | <0.001 | 9.99(7.82-12.76) | <0.001 | 4.96(4.16-5.92) | <0.001 | |
| 90 – | 11.65(10.07-13.47) | <0.001 | 17.90(13.96-22.95) | <0.001 | 6.44(5.38-7.71) | <0.001 | |
| Female sex | 0.78(0.75-0.81) | <0.001 | 0.77(0.71-0.82) | <0.001 | 0.82(0.78-0.86) | <0.001 | |
| BMI ≤ 18.5 | 1.75(1.68-1.82) | <0.001 | 2.01(1.87-2.15) | <0.001 | 1.46(1.40-1.54) | <0.001 | |
| Smoking |  |  |  |  |  |  | |
| Brinkman Index ( – 399) | 1 |  | 1 |  | 1 |  | |
| Brinkman Index (400 – 799) | 0.89(0.83-0.96) | 0.004 | 0.06(1.04-0.00) | 0.160 | 0.89(0.82-0.98) | 0.019 | |
| Brinkman Index (800 – 1199) | 0.91(0.85-0.99) | 0.020 | 0.89(0.77-1.03) | 0.109 | 0.89(0.81-0.98) | 0.013 | |
| Brinkman Index (1200 – ) | 0.84(0.77-0.90) § | <0.001 | 0.83(0.72-0.96) | 0.013 | 0.79(0.72-0.87) | <0.001 | |
| Non ambulatory status | 4.62(4.43-4.82) | <0.001 | 6.10(5.66-6.58) | <0.001 | 3.40(3.23-3.58) | <0.001 | |
| Bedsore | 2.87(2.70-3.05) | <0.001 | 3.74(3.35-4.16) | <0.001 | 2.26(2.10-2.43) | <0.001 | |
| Dialysis | 1.51(1.30-1.74) | <0.001 | — | — | 0.99(0.86-1.14) | 0.890 | |
| Immunosuppression † | 2.00(1.92-2.09) | <0.001 | — | — | 1.26(1.20-1.32) | <0.001 | |
| Residence in a nursing home or extended care facility | 2.02(1.93-2.11) | <0.001 | — | — | 1.43(1.36-1.51) | <0.001 | |
| Hospitalization during the preceding 90 days | 1.56(1.49-1.63) | <0.001 | — | — | 0.99(0.94-1.04) | 0.646 | |
| Pneumonia severity |  |  |  |  |  |  | |
| Dehydration | 3.25(3.11-3.39) | <0.001 | 3.90(3.61-4.22) | <0.001 | 2.87(2.73-3.02) | <0.001 | |
| Respiratory failure | 4.55(4.35-4.75) | <0.001 | 5.13(4.75-5.54) | <0.001 | 3.88(3.68-4.09) | <0.001 | |
| Consciousness disturbance | 5.29(5.08-5.50) | <0.001 | 6.80(6.33-7.30) | <0.001 | 4.27(4.06-4.48) | <0.001 | |
| Hypotension | 4.95(4.72-5.20) | <0.001 | 6.06(5.56-6.61) | <0.001 | 4.08(3.85-4.33) | <0.001 | |
| Critical care | 6.58(6.17-7.01) | <0.001 | 10.48(9.39-11.68) | <0.001 | 4.64(4.29-5.02) | <0.001 | |
| Comorbidities |  |  |  |  |  |  | |
| Heart failure | 2.44(2.15-2.77) | <0.001 | 2.39(1.92-2.98) | <0.001 | 2.41(2.06-2.80) | <0.001 | |
| Liver failure | 1.18(1.02-1.36) | 0.027 | 1.07(0.81-1.43) | 0.628 | 1.15(0.97-1.36) | 0.110 | |
| COPD |  |  |  |  |  |  | |
| Hugh Jones I | 1 |  | 1 |  | 1 |  | |
| Hugh Jones II | 1.34(1.12-1.59) | 0.001 | 1.37(1.03-1.82) | 0.028 | 1.24(0.99-1.54) | 0.059 | |
| Hugh Jones III | 2.13(1.81-2.52) | <0.001 | 1.59(1.19-2.12) | 0.002 | 2.20(1.79-2.70) | <0.001 | |
| Hugh Jones IV | 3.32(2.87-3.85) | <0.001 | 3.16(2.48-4.03) | <0.001 | 2.93(2.43-3.53) | <0.001 | |
| Hugh Jones V | 15.96(13.91-18.31) | <0.001 | 17.17(13.1-21.49) | <0.001 | 11.95(10.05-14.22) | <0.001 | |
| †Immunosuppression: patients who have cancer or immunodeficiency, or who use immunosuppressants. | | | | | | |  |
| Abbreviations: CAP, community-acquired pneumonia; HCAP, healthcare-associated pneumonia; BMI, body mass index; COPD, chronic obstructive pulmonary disease; MRSA, methicillin-resistant Staphylococcus aureus; MDR, multidrug-resistant. | | | | | | |  |

| Table S2. Results of univariable logistic analysis for pneumonia due to multidrug-resistant pathogens (MRSA and P. Aeruginosa) for individual potential risk factors. | | | | | | | |
| --- | --- | --- | --- | --- | --- | --- | --- |
|  | All cases of pneumonia (CAP+HCAP) |  | CAP |  | HCAP |  |  |
| Variable | Univariable-adjusted OR | P-value | Univariable-adjusted OR | P-value | Univariable-adjusted OR | P-value |  |
| Age >80 years | 1.09(1.04-1.14) | <0.001 | 1.19(1.12-1.28) | <0.001 | 0.95(0.90-1.01) | 0.110 |  |
| Female sex | 1.03(0.99-1.08) | 0.183 | 1.09(1.01-1.16) | 0.017 | 1.03(0.97-1.09) | 0.324 |  |
| BMI ≤ 18.5 | 1.80(1.72-1.88) | <0.001 | 1.79(1.67-1.92) | <0.001 | 1.68(1.58-1.77) | <0.001 |  |
| Smoking status |  |  |  |  |  |  |  |
| Brinkman Index ( – 399) | 1.0 |  | 1.0 |  | 1.0 |  |  |
| Brinkman Index (400 – 799) | 0.86(0.79-0.93) | <0.001 | 0.92(0.81-1.04) | 0.001 | 0.82(0.73-0.91) | <0.001 |  |
| Brinkman Index (800 – 1199) | 0.84(0.77-0.92) | <0.001 | 0.79(0.69-0.91) | <0.001 | 0.85(0.76-0.94) | 0.003 |  |
| Brinkman Index (1200 – ) | 0.86(0.79-0.94) | <0.001 | 0.76(0.66-0.88) | <0.001 | 0.88(0.79-0.98) | 0.022 |  |
| Non ambulatory status | 1.56(1.49-1.64) | <0.001 | 1.50(1.39-1.62) | <0.001 | 1.42(1.34-1.51) | <0.001 |  |
| Bedsore | 1.64(1.50-1.79) | <0.001 | 1.63(1.39-1.90) | <0.001 | 1.50(1.35-1.66) | <0.001 |  |
| Residence in a nursing home or extended care facility | 1.56(1.48-1.65) | <0.001 | — | — | 1.27(1.19-1.35) | <0.001 |  |
| Hospitalization during the preceding 90 days | 1.83(1.74-1.92) | <0.001 | — | — | 1.50(1.42-1.59) | <0.001 |  |
| Dialysis | 1.41(1.21-1.64) | <0.001 | — | — | 1.09(0.93-1.27) | 0.281 |  |
| Immunosuppression† | 1.08(1.02-1.13) | 0.004 | — | — | 0.69(0.65-0.73) | <0.001 |  |
| Comorbidities |  |  |  |  |  |  |  |
| Heart failure | 1.18(0.97-1.45) | 0.098 | 1.18(0.85-1.64) | 0.313 | 1.15(0.89-1.49) | 0.269 |  |
| Liver failure | 1.00(0.85-1.17) | 0.96 | 1.13(0.88-1.46) | 0.333 | 0.87(0.70-1.08) | 0.209 |  |
| COPD |  |  |  |  |  |  |  |
| Hugh Jones I | 1.0 |  | 1.0 |  | 1.0 |  |  |
| Hugh Jones II | 1.33(1.21-1.47) | <0.001 | 1.33(1.16-1.51) | <0.001 | 1.28(1.11-1.47) | <0.001 |  |
| Hugh Jones III | 1.65(1.50-1.82) | <0.001 | 1.57(1.37-1.80) | <0.001 | 1.61(1.40-1.85) | <0.001 |  |
| Hugh Jones IV | 1.88(1.72-2.05) | <0.001 | 1.87(1.65-2.12) | <0.001 | 1.69(1.49-1.92) | <0.001 |  |
| Hugh Jones V | 2.49(2.28-2.71) | <0.001 | 2.36(2.08-2.68) | <0.001fa | 2.18(1.93-2.46) | <0.001 |  |
| †Immunosuppression: patients who have cancer or immunodeficiency, or who use immunosuppressants. | | | | | | |  |
| Abbreviations: CAP, community-acquired pneumonia; HCAP, healthcare-associated pneumonia; BMI, body mass index; COPD, chronic obstructive pulmonary disease; MRSA, methicillin-resistant Staphylococcus aureus; MDR, multidrug-resistant. | | | | | | |  |
